# Licochalcone D reduces H_2_O_2_-induced SH-SY5Y cell neurotoxicity by regulating reactive oxygen species

**DOI:** 10.3389/fphar.2025.1573882

**Published:** 2025-09-18

**Authors:** Ah-Won Kwak, Seungmin Park, Ha-Na Oh, Jung-Hyun Shim, Goo Yoon, Woo-Keun Kim

**Affiliations:** 1 Center for Predictive Model Research, Division of Advanced Predictive Research, Korea Institute of Toxicology, Daejeon, Republic of Korea; 2 Department of Biomedicine, Health and Life Convergence Sciences, BK21 Four, College of Pharmacy, Mokpo National University, Muan, Republic of Korea; 3 Department of Pharmacy, College of Pharmacy, Mokpo National University, Muan, Republic of Korea; 4 The China-US (Henan) Hormel Cancer Institute, Zhengzhou, Henan, China; 5 Human and Environmental Toxicology, University of Science and Technology, Daejeon, Republic of Korea

**Keywords:** apoptosis, licochalcone D, neurotoxicity, oxidative stress, reactive oxygen species

## Abstract

Oxidative stress, one of the primary pathogenic factors in neurodegenerative diseases, plays a key role in neuronal damage via various apoptotic mechanisms. Using natural antioxidants to counteract oxidative stress may be a useful approach to slow the progression of neurodegenerative diseases. Licochalcone D (LCD), a root extract of *Glycyrrhiza inflata*, has various pharmacological activities; nonetheless, its neuroprotective effects and cellular mechanisms against oxidative damage in neuronal cells remain to be elucidated. To address this, we examined the neuroprotective effects and mechanisms of LCD in H_2_O_2_-induced cytotoxicity and neurotoxicity in the SH-SY5Y human neuroblastoma cell line. SH-SY5Y human neuroblastoma cells were differentiated using retinoic acid and subsequently treated with LCD and H_2_O_2_. Cell viability and cytotoxicity were evaluated using cell counting kit-8 and lactate dehydrogenase assays, respectively. Intracellular reactive oxygen species levels were quantified using 2′,7′-dichlorofluorescein diacetate, while mitochondrial membrane potential was assessed using 5,5′,6,6′-tetrachloro-1,1′,3,3′-tetraethylbenzimidazol-carbocyanine iodide dye. Gene expression analysis was performed by real-time qPCR, and neurite outgrowth was examined using high-content imaging. Protein expression levels were determined by Western blotting. All experiments were conducted in triplicate, and statistical analyses were performed to determine the significance of the results. LCD improved cell viability, reduced reactive oxygen species and lactate dehydrogenase levels, and protected SH-SY5Y cells from oxidative stress. High-content screening confirmed that LCD rescued the oxidative stress-induced inhibition of neurite outgrowth. LCD upregulated the mRNA expression of the neurodevelopmental genes *βIII-tubulin*, *GAP43*, *Nestin*, and *MAP2*. Mechanistically, LCD reduced p-p38 MAPK protein expression and inhibited H_2_O_2_-induced cell death by regulating the expression of apoptosis-related proteins. These findings confirm that LCD protects against H_2_O_2_-induced cytotoxicity, neurotoxicity, and p38 MAPK pathway-related apoptosis by mitigating reactive oxygen species production.

## 1 Introduction

Oxidative stress due to excessive reactive oxygen species (ROS) levels, a primary cause of nerve damage, contributes to the development of neurodegenerative diseases such as Alzheimer’s Disease, Amyotrophic Lateral Sclerosis, Huntington’s Disease, Parkinson’s Disease, and stroke (Ashok et al., 2022). Natural compounds are attracting attention as potential alternatives for treating various diseases because they have fewer side effects (Zhang et al., 2020). The antioxidant defense system plays an important role in controlling oxidative stress, and natural products can protect nerve cells from oxidative stress via various mechanisms (Jeong et al., 2021).

A small but significant portion of oxygen, approximately 4%–5%, undergoes conversion to ROS via biological reducing agents in the human body (Jomova et al., 2023). ROS, which include superoxide anions, H_2_O_2_, and OH^−^ radicals, are highly reactive (Smith et al., 2022) and play a central role in neuronal degeneration by disrupting the functions of key biomolecules, causing mitochondrial dysfunction, cellular damage, and ultimately cell death (Jomova et al., 2023). Reducing ROS production may therefore provide a promising strategy for preventing and treating these conditions. Their levels are regulated by major antioxidants, including superoxide dismutases, catalase, and glutathione peroxidases, thus maintaining homeostasis (Jiang et al., 2022; Jomova et al., 2023). Under oxidative stress, however, excessive amounts of ROS are maintained in the body owing to unstable antioxidant mechanisms, thus disrupting homeostasis (Ashok et al., 2022). H_2_O_2_, a low-reactivity radical and an ROS, participates in inflammatory processes and is cytotoxic toward various types of cells (Park, 2013; Tochigi et al., 2013). It damages cells by generating the highly reactive OH^−^ radical via the Fenton reaction, whereby it is reduced by metal ions such as iron or copper to form highly reactive and harmful hydroxyl radicals (Kumar et al., 2023). Although this renders H_2_O_2_ cytotoxic to nerve cells, antioxidants can reverse this (Jeong et al., 2021; Ashok et al., 2022).

Licorice is a traditional herbal medicine commonly used in East Asia for the treatment of various diseases (Jiang et al., 2020). Licorice extract (1 g of extract per kg body weight) has been reported to have neuroprotective effects (Zhou et al., 2017). Licochalcone D (LCD), extracted from licorice *Glycyrrhiza inflata*, is a flavonoid-based biologically active ingredient (Maharajan et al., 2021; Deng et al., 2023). LCD exerts anti-cancer effects via various pathways in cancers such as skin, oral, and lung cancer (Deng et al., 2023). Via its AMPK activity, it exerts antioxidant, anti-aging, and cardioprotective effects (Maharajan et al., 2021). Here, we examined its neuroprotective effects against H_2_O_2_-induced neurotoxicity *in vitro*.

## 2 Materials and methods

### 2.1 Materials and reagents

The reagents and materials were sourced as follows: Dulbecco’s Modified Eagle’s Medium (DMEM), trypsin/EDTA, fetal bovine serum (FBS), and penicillin/streptomycin from Gibco (Thermo Fisher Scientific, Inc., Waltham, MA, United States); phosphate-buffered saline (PBS) from Corning (Corning, NY, United States); N-Acetyl-L-cysteine (NAC), retinoic acid (RA), and dimethyl sulfoxide from Sigma-Aldrich (St. Louis, MO, United States); 4′,6-diamidino-2-phenylindole (DAPI) from Invitrogen (Thermo Fisher Scientific); anti-βIII-tubulin primary antibody from Abcam (Cambridge, United Kingdom); the Cell Counting Kit-8 from Dojindo (Kumamoto, Japan); the CytoTox 96 non-radioactive cytotoxicity assay kit and BCA Protein Assay Kit from Promega (Madison, WI, United States); RIPA lysis buffer from Rockland Immunochemicals (Limerick, PA, United States); and LCD (99.95% purity) from Selleckchem (Houston, TX, United States).

### 2.2 Cell culture, differentiation, and treatments

The SH-SY5Y human neuroblastoma cell line was obtained from the Korean Cell Bank (Seoul, Republic of Korea). The cells were cultured for 24 h in DMEM supplemented with growth medium (10% FBS and 1% penicillin/streptomycin) in a 37 °C humidified atmosphere containing 5% CO_2_. The medium was then replaced with differentiation medium (DMEM containing 1% FBS and 10 μM RA), and the cells were differentiated for 5 days. The differentiation medium was replaced after 3 days. The differentiated cells were pretreated with LCD for 3 h, and then exposed to H_2_O_2._

### 2.3 Cell counting Kit-8 (CCK-8) assay

Cell viability was assessed using a CCK-8 assay (Dojindo). SH-SY5Y cells (3.5 × 10^4^ cells/well) were cultured and differentiated in a 96-well plate, and pre-treated with 0.5, 1, or 2 μM LCD for 3 h, followed by incubation with 25 μM H_2_O_2_ for 24 h. Then, 10 μL of CCK-8 solution was added to each well, followed by incubation at 37 °C with 5% CO_2_ for 3 h. Absorbance was measured using a microplate reader (Cytation 5, BioTek, Winooski, VT, United States) at 450 nm.

### 2.4 Lactate dehydrogenase (LDH) assay

Cytotoxicity was evaluated by measuring LDH release from the cytoplasm into the culture medium upon plasma-membrane damage. The cells were plated at 3.5 × 10^4^ cells per well in a 96-well plate and cultured for 24 h, and then treated with RA diluted in medium containing 1% FBS and differentiated for 5 days. Prior to treatment with H_2_O_2_ for 24 h, the cells were pre-treated with LCD at various concentrations for 3 h. The positive controls were lysed with lysis buffer for 40 min at 37 °C in an incubator. Each sample aliquot was incubated with 50 μL of CytoTox 96 Reagent for 30 min at 25 °C in the dark. To terminate the reaction, 50 μL of Stop Solution was added to each well. Absorbance was measured at 490 nm or 492 nm within 1 h after the reaction was stopped.

### 2.5 Detection of intracellular ROS

Intracellular ROS activity was assessed using the cell-permeating fluorescent redox probe 2′,7′-dichlorofluorescein diacetate (DCFH-DA). The cells were seeded in 96-well plates at a density of 3.5 × 10^4^ cells/well and differentiated with 10 μM retinoic acid (RA) for 5 days. The cells were pretreated with vehicle or LCD for 3 h, and 20 μM DCFH-DA was added to each well for 30 min. NAC treatment was performed using the same method as LCD. Subsequently, the cells were washed with PBS and treated with 25 μM H_2_O_2_ and 50 μM tert-butyl hydroperoxide (TBHP). TBHP was utilized as a positive control to validate the experimental results. After incubation for 1 h at 37 °C, intracellular ROS levels were measured at 485 and 535 nm using a microplate reader (Cytation 5, BioTek). Fluorescence images were captured using the Operetta CLS High-Content Imaging System (PerkinElmer, Waltham, MA, USA) at 20× magnification, with the EGFP channel to visualize intracellular ROS-related fluorescence.

### 2.6 Analysis of mitochondrial membrane potential

Mitochondrial function was assessed by monitoring mitochondrial membrane potential using a fluorescent dye, 5,5′,6,6′-tetrachloro-1,1′,3,3′-tetraethylbenzimidazol-carbocyanine iodide (JC-1; Invitrogen). The cells were pretreated without LCD (control or H_2_O_2_ treatment) or with LCD at various concentrations (0.5, 1, and 2 μM) for 3 h, and then washed with PBS and incubated with JC-1 (20 μM) for 45 min at 37 °C. After incubation, the cells were washed twice with PBS and treated with 20 μM of carbonyl cyanide 3-chlorophenyolhydrazone (CCCP; Sigma) or 25 μM of H_2_O_2_ for 1 h at 37 °C. NAC treatment was performed using the same method as LCD. CCCP was employed as a positive control. Fluorescence was quantified using a microplate reader (Cytation 5). Red fluorescence intensity (indicating JC-1 aggregates) was detected at 550/600 nm (Ex/Em) and green fluorescence intensity (indicating JC-1 monomers) at 485/535 nm. Fluorescence images were acquired using the Operetta CLS High-Content Imaging System (PerkinElmer, Waltham, MA, United States) at 20× magnification, with fluorescence channels corresponding to Alexa 594 (red) and EGFP (green).

### 2.7 Quantification of intracellular ATP

Intracellular ATP levels were measured using the ATP Assay Kit (ab83355; Abcam), which employs a fluorometric enzymatic reaction to quantify ATP via phosphorylation of glycerol. For reagent preparation, Converter Mix B and Developer Mix N were each dissolved in 220 µL Buffer 23 and kept on ice. A 0.1 mM ATP standard was freshly prepared from a 10 mM stock and serially diluted (0–10 µL) in Buffer 23 to a final volume of 50 µL per well. Cells were harvested, washed with cold PBS, lysed in 100 µL Buffer 23 by pipetting, and centrifuged at 13,000 × g for 5 min at 4 °C; the supernatants were used for analysis. For each well, 1–50 µL of cell extract was adjusted to 50 µL with Buffer 23, followed by addition of 50 µL ATP reaction mix. Control reactions lacking Converter Mix B were included to assess background. After 30 min incubation at room temperature in the dark, fluorescence was measured at 535/587 nm.

### 2.8 RNA isolation, cDNA synthesis, and real-time qPCR

Cells were seeded in 100 mm cell culture plates at 4 × 10^6^ cells/mL. After 24 h, the culture medium was removed and replaced with differentiation medium containing RA. Cells were pretreated with various concentrations of LCD (up to 2 μM) then exposed to H_2_O_2_ (25 μM). Total RNA was extracted using the Monarch Total RNA Miniprep Kit (New England BioLabs, MA, United States), according to the manufacturer’s instructions. Subsequently, 1,000 ng of RNA was reverse-transcribed into cDNA using the iScript cDNA Synthesis Kit (Bio-Rad, Hercules, CA, United States). The cDNA samples were then analyzed using a StepOnePlus Real-time PCR system (Applied Biosystems, Foster City, CA, United States) with the GoTaq qPCR Master Mix (Promega). Gene expression was measured at 60 °C. The genes are listed in Table 1. To assess neuronal differentiation and development, neurite outgrowth, and structural and functional plasticity, we selected four well-established neuronal markers: βIII-tubulin (*TUBB3*), nestin (*NES*), *GAP43*, and *MAP2*. Gene expression was quantified in real-time using the comparative CT method (2^−ΔΔCT^) (Livak and Schmittgen, 2001) and was normalized to that of the endogenous control gene, *β-actin*.

**TABLE 1:** List of qRT-PCR primers.

Gene	Forward primer	Reverse primer
*Nestin*	AAC​AGC​GAC​GGA​GGT​CTC​TA	TTC​TCT​TGT​CCC​GCA​GAC​TT
*βⅢ-tubulin*	CAT​CCA​GAG​CAA​GAA​CAG​CA	CTC​GGT​GAA​CTC​CAT​CTC​GT
*GAP43*	AGG​GAG​AAG​GCA​CCA​CTA​CT	GGA​GGA​CGG​CGA​GTT​ATC​AG
*MAP2*	TGCCATCTTGGTGCCGA	CTT​GAC​ATT​ACC​ACC​TCC​AGG​T
*β-actin*	CAT​GTA​CGT​TGC​TAT​CCA​GGC	CTC​CTT​AAT​GTC​ACG​CAC​GAT

### 2.9 Neurite outgrowth assessment using high-content analysis

Cells were fixed in 4% formaldehyde in PBS for 10–15 min, and then washed three times with PBS. Permeabilization was performed using PBS containing 0.1% Triton X-100 for 15 min at room temperature. To prevent nonspecific binding, the cells were blocked for 1 h in PBS containing 10% FBS and 0.1% bovine serum albumin. They were then incubated overnight at 4 °C with the primary antibody, anti-βIII-tubulin, diluted in PBS with 0.1% Tween-20. After washing with PBS containing 0.1% Tween-20, the cells were incubated for 1 h with an Alexa Fluor 488-conjugated secondary antibody (Abcam) and 0.5 μg/mL DAPI (Invitrogen) in PBS containing 0.1% Tween-20. Fluorescence intensity was measured using the Operetta CLS High-Content Imaging System (PerkinElmer, Waltham, MA, United States) with a ×20 confocal objective. Images were analyzed using Harmony 6.1, which provides various modules for assessing neurite outgrowth.

### 2.10 Western blotting

The cells were lysed using RIPA buffer (Rockland Immunochemicals) supplemented with a protease and phosphatase inhibitor cocktail (Cell Signaling Technology, Danvers, MA, United States). Protein concentrations were determined using a BCA assay. For analysis, 20–40 μg of protein per lane was subjected to electrophoresis on 8%, 12%, or 15% SDS-PAGE gels, followed by transfer onto polyvinylidene difluoride membranes (Millipore, Bedford, MA, United States). The membranes were blocked with 3% skim milk in PBS containing 1% Tween 20 for 1–2 h at room temperature, and then incubated overnight at 4 °C with primary antibodies targeting Bax (sc-7480), Bcl-2 (sc-7382), caspase-3 (sc-56053), caspase-7 (sc-56063), GAPDH (sc-47742), PARP-1 (sc-8007), p38 (#9212), or p-p38 (#4511), all diluted 1:1000. After washing with PBS containing 1% Tween-20, the membranes were treated with HRP-conjugated secondary antibodies (goat anti-rabbit IgG-HRP or goat anti-mouse IgG-HRP) for 2 h at room temperature. Detection was performed using the WesternBright ECL substrate (Advansta, San Jose, CA, United States). Images were captured using the ChemiDoc XRS+ imaging system (Bio-Rad Laboratories).

### 2.11 Statistical analysis

The results are expressed as the mean ± standard error of the mean (SEM), derived from three or more independent experiments. Analyses were performed using SPSS 12 (IBM Corp., Armonk, NY, United States) for Windows. Statistical significance was assessed using one-way ANOVA, followed by Dunnett’s *post hoc* test. Differences were considered significant at *p* < 0.05.

## 3 Results

### 3.1 LCD protects SH-SY5Y cells against H_2_O_2_-induced cytotoxicity

**FIGURE 1:**
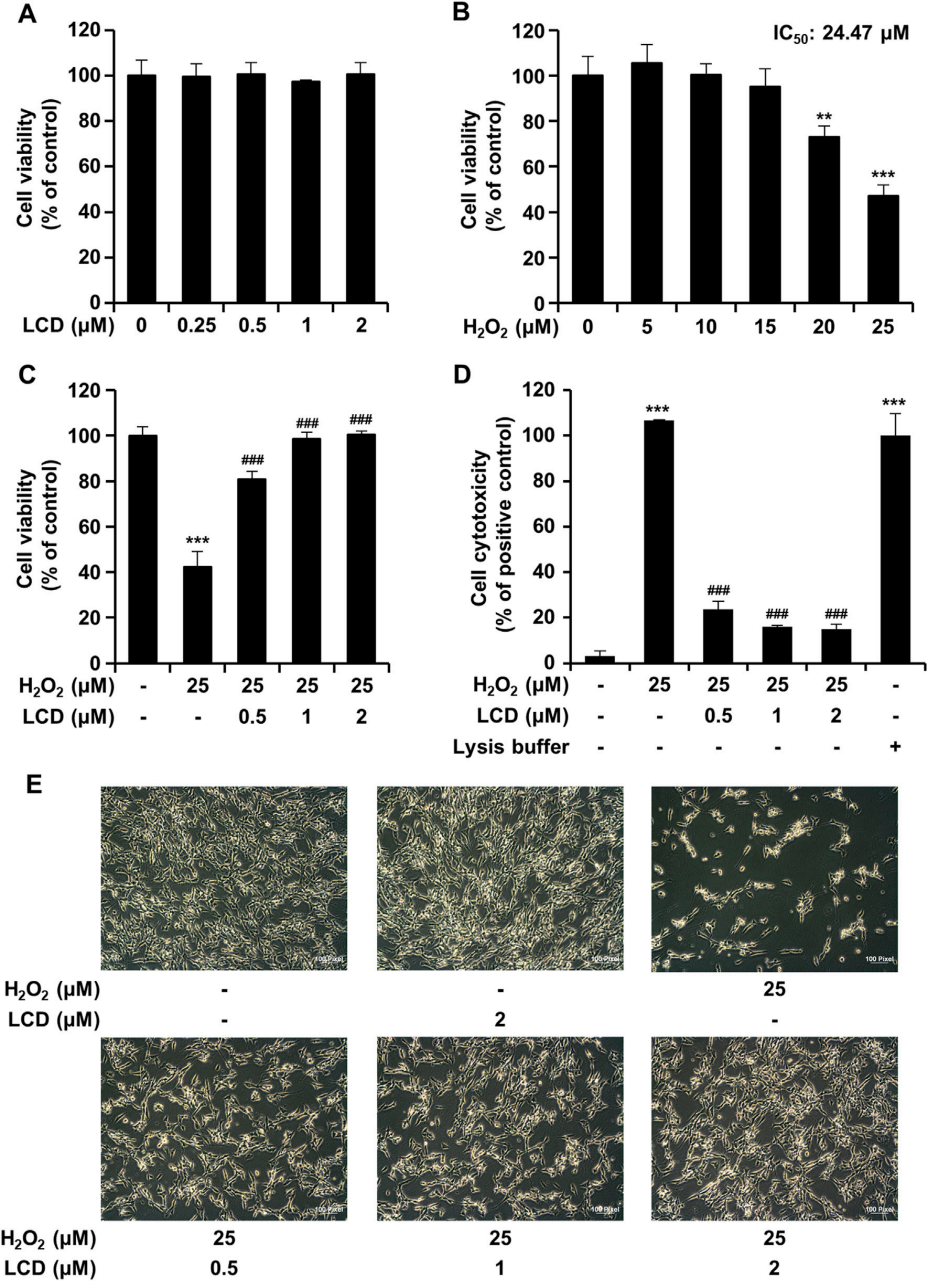
Inhibitive effects of LCD on H_2_O_2_-induced cell death. Cells were pretreated with LCD (at 0.5, 1, or 2 μM) for 3 h, and then cultured with H_2_O_2_ (25 μM) for 24 h. **(A–C)** Viability of SH-SY5Y cells was determined by CCK-8 assay. Results are presented as the mean ± SEM of three independent experiments (*n* = 5). **(D)** LDH activity was assayed using a CytoTox 96 Non-Radioactive Cytotoxicity Assay kit. **(E)** Microscopy showing the effects of LCD on H_2_O_2_-induced cytotoxicity in SH-SY5Y cells. Scale bar, 100 μm. Results are presented as the mean ± SEM of three independent experiments (*n* = 3). **p < 0.01, ***p < 0.001 vs. control group, ^###^ *p* < 0.001 vs. H_2_O_2_ treatment group.

SH-SY5Y cell growth and proliferation and LCD cytotoxicity were measured and evaluated qualitatively using CCK-8 and LDH assays. LCD did not alter cell viability (Figure 1A). The cells were differentiated in differentiation medium containing RA for 5 days and treated with H_2_O_2_ and LCD for 24 h to measure cell viability and cytotoxicity. H_2_O_2_ significantly reduced cell viability, in a concentration-dependent manner, relative to the control (Figure 1B). H_2_O_2_ inhibited cell growth, with IC_50_ values of 24.47 μM at 24 h. Therefore, in subsequent experiments, 25 μM H_2_O_2_ was used to reliably induce neurotoxicity. The LCD IC_50_ was 49.29 μM (Supplementary Figure S1). Subsequently, low-concentration LCD (0.5–2 μM), without cytotoxicity, was used. In the H_2_O_2_ treated group, LCD pretreatment at 0.5, 1, or 2 μM increased cell viability in a concentration-dependent manner (Figure 1C). Cells treated with H_2_O_2_ exhibited significantly elevated LDH activity, as did the lysis-buffer-treated positive control (Figure 1D). In the H_2_O_2_ treated group, LCD pretreatment reversed LDH activity, in a concentration-dependent manner (Figure 1D). H_2_O_2_ treatment significantly reduced cell density relative to the control and LCD treatment alone, whereas LCD pretreatment reversed the H_2_O_2_-induced reduction in cell density (Figure 1E).

### 3.2 LCD exhibits neuroprotective effects against H_2_O_2_-induced neurotoxicity

**FIGURE 2:**
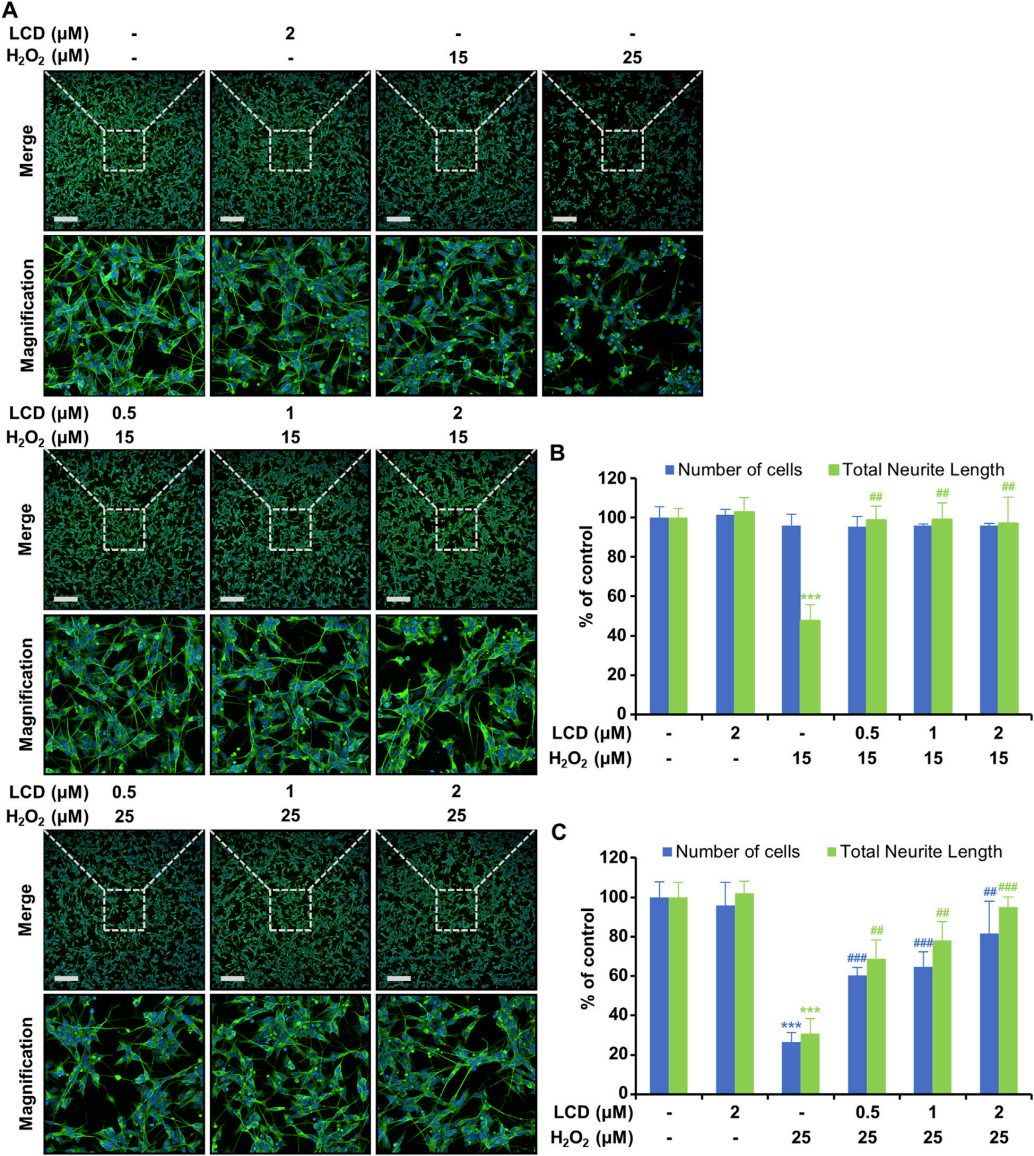
Protective effects of LCD against neurotoxicity following exposure of SH-SY5Y cells to H_2_O_2_. The cells were cultured for 5 days with retinoic acid (RA), and then pre-treated with LCD, and finally treated with H_2_O_2_ for 24 h. **(A)** Immunocytochemical fluorescence imaging of SH-SY5Y cells using anti-βIII-tubulin antibody (green) and DAPI (blue) staining. **(B,C)** Fluorescence intensity to quantify cell count and total neurite length. The results are presented as mean ± SEM (*N* = 3). ***p < 0.001 vs. the untreated control group; ^##^ *p* < 0.01 and ^###^ *p* < 0.001 vs. the H_2_O_2_ treatment group.

To assess the neuroprotective effects of LCD, neurite outgrowth was measured using a high-content screening device. At a non-cytotoxic H_2_O_2_ concentration (15 μM), there was no significant reduction in cell count (Figure 1B), although neurite outgrowth length was reduced by >50% (Figure 2B). The LCD and H_2_O_2_ co-treatment group exhibited results similar to the control in terms of both cell count and neurite outgrowth length (Figure 2B). H_2_O_2_ at 25 μM reduced both the cell count and neurite growth length (Figure 2C), similar to its effects on cell viability (Figure 1). Under LCD and H_2_O_2_ co-treatment, both cell count and neurite growth length increased in proportion to the LCD concentration (Figure 2C).

**FIGURE 3:**
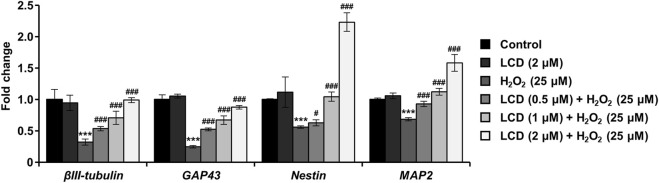
Impacts of LCD on neurodevelopmental genes in differentiated SH-SY5Y cells with H_2_O_2_-induced neurotoxicity. The expression of neurodevelopmental genes (*βIII-tubulin*, *GAP43*, *Nestin*, and *MAP2*) was analyzed using qRT-PCR, using β-actin as an internal control (*N* = 3). ***p < 0.001 vs. the control group; ^#^ *p* < 0.05, ^###^ *p* < 0.001 vs. with the H_2_O_2_ treatment group.

We investigated four genes directly associated with neural differentiation, development, and neurite outgrowth (*βIII-tubulin*, *GAP43*, *Nestin*, and *MAP2*). H_2_O_2_ treatment reduced their expression (Figure 3), with *βIII-tubulin* and *GAP43* expression declining by >50%. Conversely, in the LCD treatment, the expression of these genes was similar to that in the untreated control. Under LCD and H_2_O_2_ co-treatment, the expression of the four genes increased with the LCD concentration. Following co-treatment with H_2_O_2_ and 2 μM LCD, *βIII-tubulin* and *GAP43* expression was similar to that in the untreated control, whereas that of *NES* and *MAP2* was elevated, by 2.23-fold and 1.58-fold, respectively, relative to that in the untreated control.

### 3.3 NAC mitigates H_2_O_2_-induced ROS production and neurotoxicity in SH-SY5Y cells

**FIGURE 4:**
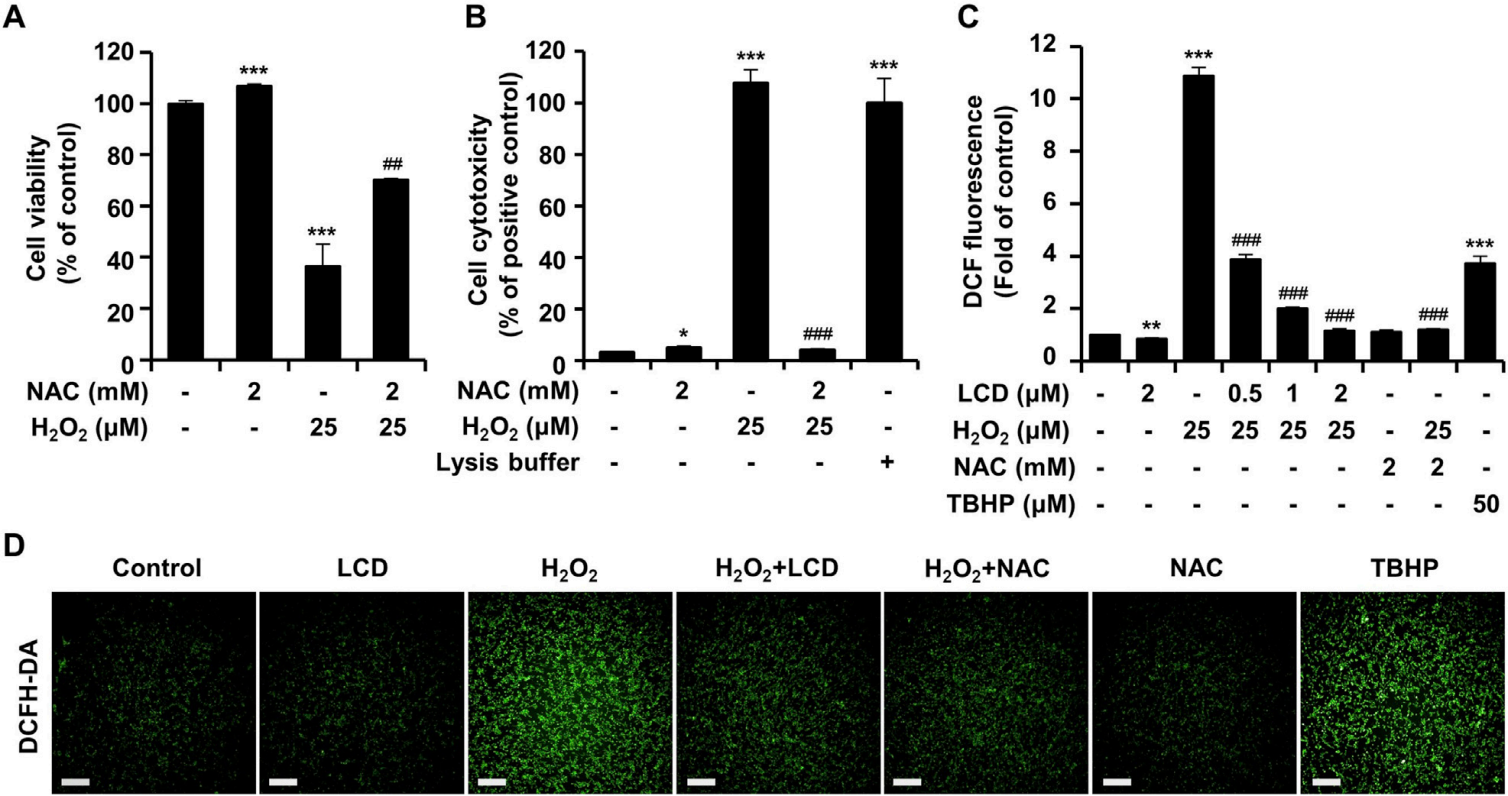
Effects of NAC and LCD pretreatment on the H_2_O_2_-induced decline in cell viability, cytotoxicity and ROS generation. Differentiated SH-SY5Y cells were pretreated with 2 mM NAC for 3 h, followed by exposure to 25 μM H_2_O_2_ for 24 h, to assess **(A)** cell viability via CCK-8 assay and **(B)** cytotoxicity via LDH assay. **(C)** For ROS measurement, differentiated cells were pretreated with 2 mM NAC or 2 μM LCD for 3 h, and then treated with 25 μM H_2_O_2_ or 50 μM TBHP for 1 h. Intracellular ROS levels, quantified using the DCFH-DA assay. **(D)** Representative fluorescence images of DCFH-DA staining (green). Scale bar: 200 μm. Experiments were performed in triplicate (*n* = 3). The results are presented as mean ± SEM; *p < 0.05, **p < 0.01, and ***p < 0.001 vs. the control group; ^##^ *p* < 0.01, and ^###^ *p* < 0.001 vs. the H_2_O_2_-treated group.

To assess whether the H_2_O_2_-induced cytotoxicity and neurotoxicity in SH-SY5Y cells is due to ROS production, we evaluated cell viability and neurite outgrowth with and without the antioxidant NAC. When treated with NAC, the results were comparable with those in the untreated control, whereas H_2_O_2_ treatment alone reduced cell viability to <50% (Figure 4A). NAC and H_2_O_2_ co-treatment led to higher cell viability than treatment with H_2_O_2_ alone (Figure 4A). Cytotoxicity was minimal in the untreated control, the NAC-only treatment, and the LCD + H_2_O_2_ co-treatment (Figure 4B). In contrast, H_2_O_2_ alone caused high cytotoxicity, comparable to that in the positive control (lysis buffer) (Figure 4B). LCD treatment alone had no effect on intracellular ROS production (Figure 4C). In contrast, intracellular ROS accumulation was substantially higher following treatment with H_2_O_2_ alone than in the positive TBHP control (Figure 4C). Co-treatment of LCD and NAC achieved neuroprotective and ROS-reducing effects at concentrations at which the CCK-8 assay confirmed no cytotoxicity in single and combined treatments (Supplementary Figure S2). This co-treatment did not result in synergistic effects that differed significantly from their effects when administered separately. Nevertheless, comprehensive dose-response studies and the application of formal synergy evaluation methods will be essential to rigorously assess the potential additive or synergistic interactions of LCD and NAC co-treatment.

**FIGURE 5:**
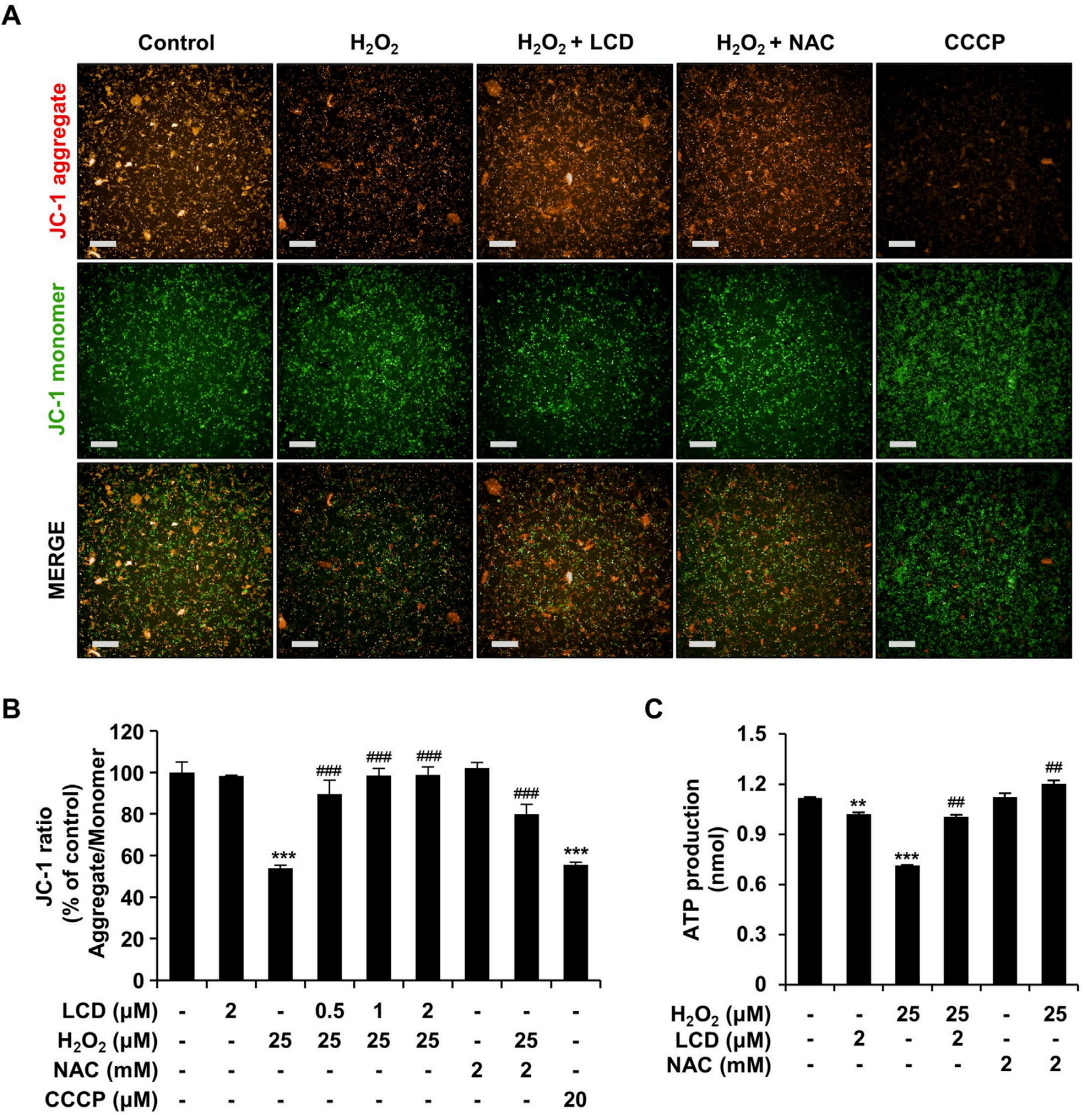
Mitigation of H_2_O_2_-induced loss of mitochondrial-membrane potential and depletion of ATP by NAC and LCD pretreatment. Differentiated SH-SY5Y cells were pretreated with 2 mM NAC or 2 μM LCD for 3 h, and then treated with 25 μM H_2_O_2_ for 24 h. Mitochondrial membrane potential was assessed using the JC-1 assay, with CCCP as a positive control. JC-1 aggregates (red) were fluoresced at 560/635 nm and the JC-1 monomer (green) at 485/535 nm. **(A)** Representative JC-1 fluorescence images. Scale bar: 200 μm. **(B)** Quantitative analysis of the red/green fluorescence intensity ratio. Experiments were performed in triplicate (*n* = 3). **(C)** Intracellular ATP levels, measured using an ATP assay kit (ab83355). The results are presented as mean ± SEM (*n* = 3). **p < 0.01, and ***p < 0.001 vs. the control group; ^##^ *p* < 0.01 and ^###^ *p* < 0.001 vs. the H_2_O_2_-treated group.

**FIGURE 6:**
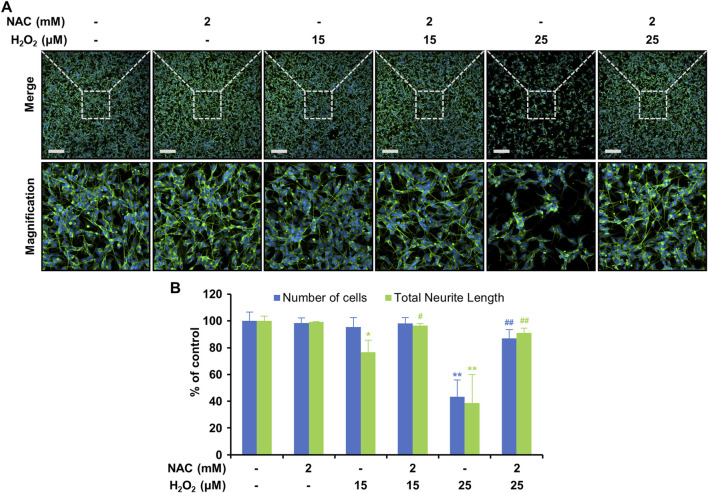
Effects of NAC on neurite outgrowth in differentiated SH-SY5Y cells pretreated with H_2_O_2_. Cells were pretreated with 2 mM NAC for 3 h and then with 25 μM H_2_O_2_ for 24 h. **(A)** Fluorescence imaging of immunostaining. Scale bar, 200 μM. **(B)** Quantification of cell count and total neurite length via immunostaining (*n* = 3). The results are expressed as mean ± SEM (*N* = 3). *p < 0.05 and **p < 0.01 vs. the control; ^#^ *p* < 0.05 and ^##^ *p* < 0.01 vs. the H_2_O_2_-treated group.

Oxidative-stress-induced mitochondrial dysfunction is an important factor in neurodegenerative disorders (Ashok et al., 2022; Jiang et al., 2022). We performed JC-1 analysis to examine the effects of H_2_O_2_ and LCD on mitochondria. Neither LCD nor NAC alone significantly altered mitochondrial membrane potential. However, the ratio of red to green fluorescence decreased following treatment with H_2_O_2_ (Figures 5A,B), whereas H_2_O_2_ and LCD co-treatment increased this ratio in an LCD-concentration-dependent manner; a similar increase was observed under H_2_O_2_ and NAC co-treatment. ATP-production analysis, used to evaluate the effects of mitochondrial functional changes on ATP levels, revealed that LCD and NAC significantly restored the ATP levels reduced by H_2_O_2_ (Figure 5C). These results indicate that LCD reduces H_2_O_2_-induced intracellular ROS production and restores mitochondrial function in SH-SY5Y cells. Under co-treatment with H_2_O_2_, LCD reduced ROS production in a concentration-dependent manner (Figure 4C). NAC similarly reduced ROS production (Figure 4C). Neurite outgrowth length was 99.22% under NAC treatment, 76.60% under 15 μM H_2_O_2_ treatment, and 96.48% under NAC (2 mM) and H_2_O_2_ (15 μM) co-treatment, relative to neurite outgrowth length in the untreated control. Similarly, it was 38.61% under 25 μM H_2_O_2_ treatment and 91.04% under NAC (2 mM) and H_2_O_2_ (25 μM) co-treatment (Figures 6A,B). The cell count was not significantly reduced following treatment with NAC, 15 μM H_2_O_2_, or co-treatment of NAC (2 mM) with either 15 μM or 25 μM H_2_O_2_. However, treatment with 25 μM H_2_O_2_ alone significantly reduced the cell count (Figures 6A,B).

### 3.4 LCD inhibits H_2_O_2_-induced apoptosis

**FIGURE 7:**
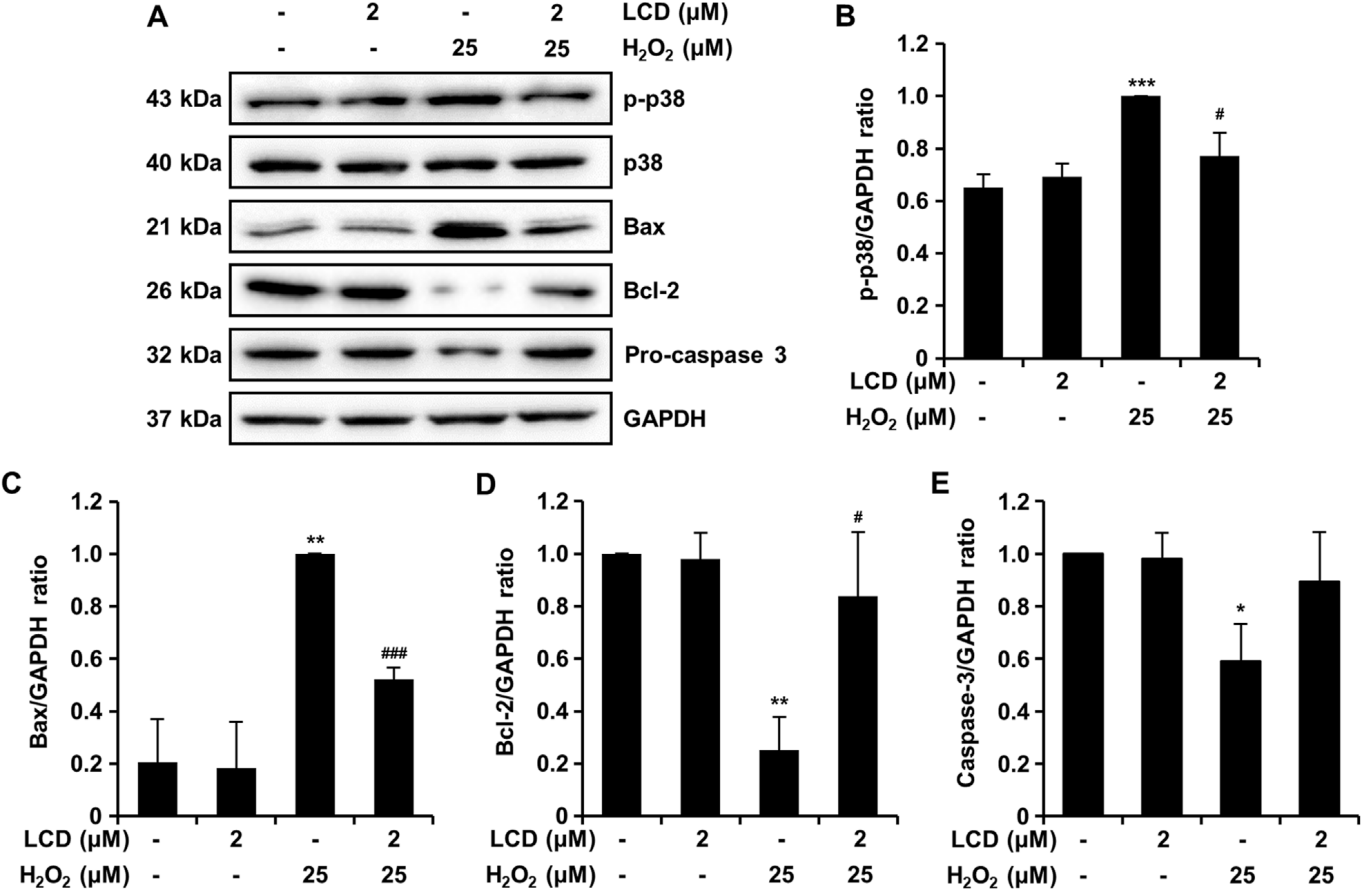
Western blotting was performed to quantify apoptosis-related protein expression in SH-SY5Y cells. **(A)** Proteins were separated by gel electrophoresis, and apoptosis-related protein expression was evaluated, using GAPDH as an internal control. **(B–E)** Expression of apoptosis-related proteins, with the band intensity ratio expressed as mean ± SEM (*N* = 3). *p < 0.05, **p < 0.01, and ***p < 0.001 vs. the control; ^#^ *p* < 0.05; ^###^ *p* < 0.001 vs. the H_2_O_2_-treated group.

To evaluate the underlying mechanisms of action of LCD, we quantified the expression of H_2_O_2_-induced apoptosis-related proteins via Western blotting. H_2_O_2_ increased p-p38 expression, whereas LCD pretreatment reduced it (Figures 7A,B). H_2_O_2_ increased the expression of the pro-apoptotic protein Bax (Figures 7A,C) and reduced that of the anti-apoptotic protein Bcl-2 (Figures 7A,D). However, these effects were reversed under LCD and H_2_O_2_ co-treatment: Bax expression was reduced (Figures 7A,C) whereas that of Bcl-2 was increased (Figures 7A,D). H_2_O_2_ alone reduced pro-caspase 3 expression, whereas co-treatment with LCD rescued it (Figures 7A,E). These results suggest that LCD effectively inhibits H_2_O_2_-induced apoptosis.

## 4 Discussion

Using SH-SY5Y cells, we examined the neuroprotective effects of LCD and its potential mechanisms of action in mitigating H_2_O_2_-induced oxidative stress. LCD exposure for 24 h did not induce significant cytotoxicity in SH-SY5Y cells (Figure 1A). This is consistent with prior findings that LCD at concentrations of ≤22.6 μM did not significantly reduce the viability of human bone marrow mesodermal stem cells (Maharajan et al., 2021). H_2_O_2_ exposure, in contrast, caused concentration-dependent cytotoxicity, reducing cell viability to <50% at 25 μM H_2_O_2_ (Figure 1B). Based on our findings, LCD reversed the H_2_O_2_-induced reduction in cell viability (Figure 1C).

The developing nervous system is highly sensitive to the influence of various chemicals (Crofton et al., 2012). Neurocytoma cell lines have been used *in vitro* to confirm developmental neurotoxicity, as they are abundant, easy to culture, and exhibit homogenous populations (Radio and Mundy, 2008). Neural cell lines can be differentiated into cells with distinct neural characteristics by adding growth factors or by withholding serum (Shipley et al., 2016). We therefore treated the SH-SY5Y cell line with RA to induce differentiation into a neuronal cell line. Neurite outgrowth, a hallmark of neurodevelopment, is a key factor used to assess developmental neurotoxicity (Ryan et al., 2016). Using this differentiated SH-SY5Y cell line, we confirmed the neuroprotective effects of LCD. H_2_O_2_ caused developmental neurotoxicity (DNT) at non-cytotoxic concentrations, and pretreatment with LCD mitigated this DNT-related cytotoxicity (Figure 2). Similarly, LCD mitigated H_2_O_2_ cytotoxicity. This suggests that LCD may be effective in alleviating oxidative stress-induced DNT.

The observed reduction in neurite length indicates inhibition of neurodevelopment and altered expression of the related genes (Oh et al., 2022). *βIII-tubulin*, which is constitutively expressed in all neurons, plays important roles in neuronal structure and function (Jiang and Oblinger, 1992). *Nestin* participates in organizing the neuronal cytoskeleton and is used as a neuroepithelial stem cell marker (Deng and Poretz, 2003). *MAP2*, a widely used neural marker, plays an important role in neuronal morphogenetic processes such as neuronal initiation by interacting with both microtubules and F-actin (Dehmelt and Halpain, 2005). *GAP43*, which plays an important role in axon elongation, synaptogenesis, and neuronal sprouting, is downregulated in the brains of patients with Parkinson’s Disease (Chung et al., 2020). Our findings reveal that H_2_O_2_ exposure induced developmental neurotoxicity through pathways related to cytoskeleton regulation, neurodevelopment and maturation, as indicated by the reduced expression of *βIII-tubulin*, *Nestin*, *MAP2*, *GAP43* (Figure 3). Consequently, to advance the clinical application of LCD, clarifying whether increasing the levels of neurite-outgrowth markers improves synapse formation and neuroplasticity and, thus, restores electrophysiological function will be important (Reese and Drapeau, 1998; Fauth and Tetzlaff, 2016; Yacoub et al., 2025). The concentration of LCD used here is within the effective range reported by previous *in vitro* neuronal cell studies. However, based on data from animal studies, the actual LCD concentrations reached in the brain may be lower. Therefore, increasing blood–brain barrier (BBB) permeability and improving pharmacokinetic properties are essential for the clinical application of LCD. Strategies to enhance BBB permeability using various delivery vehicles and physical methods, such as nanoparticle carriers and targeting ligands, can be considered. However, the physicochemical properties of LCD, including its low molecular weight, high lipid solubility, and low polarity, are favorable for BBB penetration. Therefore, if its limitations can be addressed, LCD may hold significant potential as a therapeutic agent for central nervous system diseases (Pardridge, 2012; Choudhari et al., 2021). Moreover, these findings provide evidence that LCD protects against developmental neurotoxicity by rescuing the H_2_O_2_-induced inhibition of gene expression (Figure 3). However, as polyphenol compounds such as LCD can cause off-target effects owing to their promiscuity, future studies using siRNA or selective inhibitors may be required to validate the target pathways.

H_2_O_2_ treatment induces excess ROS production (Chidawanyika and Supattapone, 2021). To determine the effectiveness of LCD in alleviating H_2_O_2_-induced oxidative stress in SH-SY5Y cells, we compared its effects with those of NAC, a commonly used antioxidant. NAC pretreatment rescued the H_2_O_2_-induced reduction in cell viability (Figure 4A) and reduced the H_2_O_2_-induced release of LDH (Figure 4B), achieving similar effects as LCD pretreatment. Both NAC and LCD pretreatment rescued the excessive ROS production induced by H_2_O_2_ (Figure 4C), exhibiting similar effectiveness. We used NAC as a control to investigate how LCD achieves its neuroprotective effects by attenuating oxidative stress. The efficacy of LCD can be comprehensively validated through further studies, including those utilizing Food and Drug Administration-approved neuroprotective agents as comparators. High intracellular ROS levels can damage cellular organelles such as mitochondria (Redza-Dutordoir and Averill-Bates, 2016). Here, H_2_O_2_ induced mitochondrial depolarization, which was reversed by LCD or NAC, with LCD being slightly more effective than NAC (Figure 5B). Increased intracellular ROS production is linked to the development of pathologies such as cancer and diabetes, as well as stroke and neurodegenerative diseases such as Parkinson’s Disease and Alzheimer’s Disease (Redza-Dutordoir and Averill-Bates, 2016; Collin, 2019; Ashok et al., 2022). Our findings confirm the effectiveness of NAC against ROS-induced neurotoxicity; NAC pretreatment effectively rescued the reduction in neurite outgrowth at non-toxic H_2_O_2_ concentrations, with similar results at toxic concentrations (Figure 6). This is consistent with the findings for LCD pretreatment, thus confirming that LCD acts like NAC to inhibit neurotoxicity by reducing ROS production. These results suggest that LCD may protect neurons from oxidative stress-induced mitochondrial dysfunction and neurotoxicity. Moreover, comprehensive dose-response studies and the application of formal synergy evaluation methods will be essential to rigorously assess the potential additive or synergistic interactions of LCD and NAC co-treatment (Supplementary Figure S2). This remains an open question for future pharmacological assessment.

Macromolecules such as H_2_O_2_ can generate excess ROS, which can lead to cell death through processes such as necrosis and apoptosis (Chidawanyika and Supattapone, 2021). In Parkinson’s Disease models, oxidative stress activates the p38 MAPK pathway, ultimately leading to apoptosis (Tong et al., 2018). Here, H_2_O_2_ treatment induced the phosphorylation of p38, which was reduced by LCD (Figure 7B). The Bcl-2 protein family regulates cell death by mediating direct binding between pro-apoptotic and anti-apoptotic proteins (Carrington et al., 2017). The anti-apoptotic proteins (guardians), including A1, Bcl-2, Bcl-XL, Bcl-W, and Mcl-1, promote cell survival by inhibiting mitochondrial outer-membrane permeation by BAX and BAK, downstream pro-apoptotic proteins (effectors) (Carrington et al., 2017). Here, H_2_O_2_ promoted Bax expression and reduced that of Bcl-2, and LCD reversed these effects (Figures 7C,D). Most apoptotic pathways lead to the activation of caspases, and apoptotic caspases comprise upstream initiators and downstream effectors (Redza-Dutordoir and Averill-Bates, 2016). Here, LCD effectively inhibited the expression of H_2_O_2_-induced cleaved caspase-3 (Figure 7E). H_2_O_2_-induced oxidative stress activated the p38 MAPK apoptosis-related pathway, and LCD reversed this activation. The oxidative stress-reducing and cell viability-enhancing effects of LCD are consistent with findings reported for patient-derived induced pluripotent stem cells (Oh et al., 2023), supporting the neuroprotective potential and clinical applicability of LCD (Figures 1C,D, 4). In SH-SY5Y cells, LCD significantly reduced H_2_O_2_-induced p38 MAPK pathway-related apoptosis, ROS accumulation, loss of mitochondrial membrane potential, and neurotoxicity. These antioxidant and apoptotic effects may be related to the inhibition of the ROS-induced MAPK kinase cascade and subsequent downstream caspase cascade. Furthermore, considering the anti-inflammatory activity of compounds in the licochalcone family (Furusawa et al., 2009; Park et al., 2011), LCD may exhibit protective effects in various neurotoxic environments, including those characterized by oxidative stress or inflammatory toxic stimuli; this requires further verification. The interplay between ROS signals and upstream regulators of the p38 MAPK pathway represents a potentially important focus for future mechanistic investigations of the effects of LCD (Park et al., 2015; Deng et al., 2023; Lee et al., 2025). Validating these interactions and effects in chronic or repetitive oxidative stress models will provide further insight into the therapeutic potential of LCD. The current *in vitro* findings offer valuable mechanistic insights; however, the absence of *in vivo* validation remains a key limitation, underscoring the necessity for future studies to establish the pharmacological significance within a physiological context.

## Data Availability

The original contributions presented in the study are included in the article/Supplementary Material, further inquiries can be directed to the corresponding author.

## References

[B1] Ashok A. Andrabi S. S. Mansoor S. Kuang Y. Kwon B. K. Labhasetwar V. (2022). Antioxidant therapy in oxidative stress-induced neurodegenerative diseases: role of nanoparticle-based drug delivery systems in clinical translation. Antioxidants (Basel) 11, 408. 10.3390/antiox11020408 35204290 PMC8869281

[B2] Carrington E. M. Zhan Y. Brady J. L. Zhang J. G. Sutherland R. M. Anstee N. S. (2017). Anti-apoptotic proteins BCL-2, MCL-1 and A1 summate collectively to maintain survival of immune cell populations both *in vitro* and *in vivo* . Cell Death Differ. 24, 878–888. 10.1038/cdd.2017.30 28362427 PMC5423112

[B3] Chidawanyika T. Supattapone S. (2021). Hydrogen peroxide-induced cell death in mammalian cells. J. Cell Signal 2, 206–211. 10.33696/signaling.2.052 35079745 PMC8786222

[B4] Choudhari M. Hejmady S. Narayan Saha R. N. Damle S. Singhvi G. Alexander A. (2021). Evolving new-age strategies to transport therapeutics across the blood–brain-barrier. Int. J. Pharm. 599, 120351. 10.1016/j.ijpharm.2021.120351 33545286

[B5] Chung D. Shum A. Caraveo G. (2020). GAP-43 and BASP1 in axon regeneration: implications for the treatment of neurodegenerative diseases. Front. Cell Dev. Biol. 8, 567537. 10.3389/fcell.2020.567537 33015061 PMC7494789

[B6] Collin F. (2019). Chemical basis of reactive oxygen species reactivity and involvement in neurodegenerative diseases. Int. J. Mol. Sci. 20, 2407. 10.3390/ijms20102407 31096608 PMC6566277

[B7] Crofton K. M. Mundy W. R. Shafer T. J. (2012). Developmental neurotoxicity testing: a path forward. Congenit. Anom. (Kyoto) 52, 140–146. 10.1111/j.1741-4520.2012.00377.x 22925214

[B8] Dehmelt L. Halpain S. (2005). The MAP2/Tau family of microtubule-associated proteins. Genome Biol. 6, 204. 10.1186/gb-2004-6-1-204 15642108 PMC549057

[B9] Deng W. Poretz R. D. (2003). Oligodendroglia in developmental neurotoxicity. Neurotoxicology 24, 161–178. 10.1016/S0161-813X(02)00196-1 12606289

[B10] Deng N. Qiao M. Li Y. Liang F. Li J. Liu Y. (2023). Anticancer effects of licochalcones: a review of the mechanisms. Front. Pharmacol. 14, 1074506. 10.3389/fphar.2023.1074506 36755942 PMC9900005

[B11] Fauth M. Tetzlaff C. (2016). Opposing effects of neuronal activity on structural plasticity. Front. Neuroanat. 10, 75. 10.3389/fnana.2016.00075 27445713 PMC4923203

[B12] Furusawa J.-I. Funakoshi-Tago M. Tago K. Mashino T. Inoue H. Sonoda Y. (2009). Licochalcone A significantly suppresses LPS signaling pathway through the inhibition of NF-kappaB p65 phosphorylation at serine 276. Cell. Signal. 21, 778–785. 10.1016/j.cellsig.2009.01.021 19168128

[B13] Jeong Y. H. Kim T. I. Oh Y. C. Ma J. Y. (2021). Chrysanthemum indicum prevents hydrogen peroxide-induced neurotoxicity by activating the TrkB/Akt signaling pathway in hippocampal neuronal cells. Nutrients 13, 3690. 10.3390/nu13113690 34835946 PMC8618340

[B14] Jiang Y. Q. Oblinger M. M. (1992). Differential regulation of beta III and other tubulin genes during peripheral and central neuron development. J. Cell Sci. 103, 643–651. 10.1242/jcs.103.3.643 1478962

[B15] Jiang M. Zhao S. Yang S. Lin X. He X. Wei X. (2020). An “essential herbal medicine”-licorice: a review of phytochemicals and its effects in combination preparations. J. Ethnopharmacol. 249, 112439. 10.1016/j.jep.2019.112439 31811935

[B16] Jiang Y. Kang Y. Liu J. Yin S. Huang Z. Shao L. (2022). Nanomaterials alleviating redox stress in neurological diseases: mechanisms and applications. J. Nanobiotechnology 20, 265. 10.1186/s12951-022-01434-5 35672765 PMC9171999

[B17] Jomova K. Raptova R. Alomar S. Y. Alwasel S. H. Nepovimova E. Kuca K. (2023). Reactive oxygen species, toxicity, oxidative stress, and antioxidants: chronic diseases and aging. Arch. Toxicol. 97, 2499–2574. 10.1007/s00204-023-03562-9 37597078 PMC10475008

[B18] Kumar U. Shrivastava A. De A. K. Pai M. R. Sinha I. J. C. S. (2023). Fenton reaction by H_2_O_2_ produced on a magnetically recyclable Ag/CuWO_4_/NiFe_2_O_4_ photocatalyst. Catal. Sci. Technol. 13, 2432–2446. 10.1039/d3cy00102d

[B19] Lee S.-O. Joo S. H. Cho S.-S. Yoon G. Choi Y. H. Park J. W. (2025). Licochalcone D exerts antitumor activity in human colorectal cancer cells by inducing ROS generation and phosphorylating JNK and p38 MAPK. Biomol. Ther. Seoul. 33, 344–354. 10.4062/biomolther.2024.123 39933827 PMC11893492

[B20] Livak K. J. Schmittgen T. D. (2001). Analysis of relative gene expression data using real-time quantitative PCR and the 2(-Delta Delta C(T)) method. Methods 25, 402–408. 10.1006/meth.2001.1262 11846609

[B21] Maharajan N. Ganesan C. D. Moon C. Jang C. H. Oh W. K. Cho G. W. (2021). Licochalcone D ameliorates oxidative stress-induced senescence via AMPK activation. Int. J. Mol. Sci. 22, 7324. 10.3390/ijms22147324 34298945 PMC8304008

[B22] Oh H. N. Park S. Lee S. Chun H. S. Shin W. H. Kim W. K. (2022). *In vitro* neurotoxicity evaluation of biocidal disinfectants in a human neuron-astrocyte co-culture model. Toxicol. Vitro 84, 105449. 10.1016/j.tiv.2022.105449 35872077

[B23] Oh M. Nam J. Baek A. Seo J.-H. Chae J.-I. Lee S.-Y. (2023). Neuroprotective effects of licochalcone D in oxidative-stress-induced primitive neural stem cells from Parkinson’s disease patient-derived iPSCs. Biomedicines 11, 228. 10.3390/biomedicines11010228 36672736 PMC9856162

[B24] Pardridge W. M. (2012). Drug transport across the blood–brain barrier. J. Cereb. Blood Flow. Metab. 32, 1959–1972. 10.1038/jcbfm.2012.126 22929442 PMC3494002

[B25] Park W. H. (2013). The effects of exogenous H2O2 on cell death, reactive oxygen species and glutathione levels in calf pulmonary artery and human umbilical vein endothelial cells. Int. J. Mol. Med. 31, 471–476. 10.3892/ijmm.2012.1215 23254439

[B26] Park G.-M. Jun J.-G. Kim J.-K. (2011). Anti-inflammatory effect of licochalcone E, a constituent of licorice, on lipopolysaccharide-induced inflammatory responses in murine macrophages. J. Life Sci. 21, 656–663. 10.5352/JLS.2011.21.5.656

[B27] Park M.-R. Kim S.-G. Cho I. A. Oh D. Kang K.-R. Lee S.-Y. (2015). Licochalcone-A induces intrinsic and extrinsic apoptosis via ERK1/2 and p38 phosphorylation-mediated TRAIL expression in head and neck squamous carcinoma FaDu cells. Food Chem. Toxicol. 77, 34–43. 10.1016/j.fct.2014.12.013 25572524 PMC4522946

[B28] Radio N. M. Mundy W. R. (2008). Developmental neurotoxicity testing *in vitro*: models for assessing chemical effects on neurite outgrowth. Neurotoxicology 29, 361–376. 10.1016/j.neuro.2008.02.011 18403021

[B29] Redza-Dutordoir M. Averill-Bates D. A. (2016). Activation of apoptosis signalling pathways by reactive oxygen species. Biochim. Biophys. Acta 1863, 2977–2992. 10.1016/j.bbamcr.2016.09.012 27646922

[B30] Reese D. Drapeau P. (1998). Neurite growth patterns leading to functional synapses in an identified embryonic neuron. J. Neurosci. 18, 5652–5662. 10.1523/JNEUROSCI.18-15-05652.1998 9671656 PMC6793058

[B31] Ryan K. R. Sirenko O. Parham F. Hsieh J. H. Cromwell E. F. Tice R. R. (2016). Neurite outgrowth in human induced pluripotent stem cell-derived neurons as a high-throughput screen for developmental neurotoxicity or neurotoxicity. Neurotoxicology 53, 271–281. 10.1016/j.neuro.2016.02.003 26854185

[B32] Shipley M. M. Mangold C. A. Szpara M. L. (2016). Differentiation of the SH-SY5Y human neuroblastoma cell line. J. Vis. Exp. 108, 53193. 10.3791/53193 26967710 PMC4828168

[B33] Smith A. N. Shaughness M. Collier S. Hopkins D. Byrnes K. R. (2022). Therapeutic targeting of microglia mediated oxidative stress after neurotrauma. Front. Med. (Lausanne) 9, 1034692. 10.3389/fmed.2022.1034692 36405593 PMC9671221

[B34] Tochigi M. Inoue T. Suzuki-Karasaki M. Ochiai T. Ra C. Suzuki-Karasaki Y. (2013). Hydrogen peroxide induces cell death in human TRAIL-resistant melanoma through intracellular superoxide generation. Int. J. Oncol. 42, 863–872. 10.3892/ijo.2013.1769 23314732

[B35] Tong H. Zhang X. Meng X. Lu L. Mai D. Qu S. (2018). Simvastatin inhibits activation of NADPH Oxidase/p38 MAPK pathway and enhances expression of antioxidant protein in Parkinson disease models. Front. Mol. Neurosci. 11, 165. 10.3389/fnmol.2018.00165 29872377 PMC5972184

[B36] Yacoub M. Iqbal F. Khan Z. Syeda A. Lijnse T. Syed N. I. (2025). Neuronal growth patterns and synapse formation are mediated by distinct activity-dependent mechanisms. Sci. Rep. 15, 17338. 10.1038/s41598-025-00806-9 40389417 PMC12089460

[B37] Zhang L. Song J. Kong L. Yuan T. Li W. Zhang W. (2020). The strategies and techniques of drug discovery from natural products. Pharmacol. Ther. 216, 107686. 10.1016/j.pharmthera.2020.107686 32961262

[B38] Zhou Y.-Z. Zhao F.-F. Gao L. Du G.-H. Zhang X. Qin X.-M. (2017). Licorice extract attenuates brain aging of d-galactose induced rats through inhibition of oxidative stress and attenuation of neuronal apoptosis. RSC Adv. 7, 47758–47766. 10.1039/C7RA07110H

