# Correction: Licochalcone D reduces H_2_O_2_-induced SH-SY5Y cell neurotoxicity by regulating reactive oxygen species

**DOI:** 10.3389/fphar.2025.1721860

**Published:** 2026-01-07

**Authors:** Ah-Won Kwak, Seungmin Park, Ha-Na Oh, Jung-Hyun Shim, Goo Yoon, Woo-Keun Kim

**Affiliations:** 1 Center for Predictive Model Research, Division of Advanced Predictive Research, Korea Institute of Toxicology, Daejeon, Republic of Korea; 2 Department of Biomedicine, Health and Life Convergence Sciences, BK21 Four, College of Pharmacy, Mokpo National University, Muan, Republic of Korea; 3 Department of Pharmacy, College of Pharmacy, Mokpo National University, Muan, Republic of Korea; 4 The China-US (Henan) Hormel Cancer Institute, Zhengzhou, Henan, China; 5 Human and Environmental Toxicology, University of Science and Technology, Daejeon, Republic of Korea

**Keywords:** apoptosis, licochalcone D, neurotoxicity, oxidative stress, reactive oxygen species

In the published article, there was an error in **Affiliation 5**. Instead of “Department of Pharmacy, College of Pharmacy, Mokpo National University, Muan, Republic of Korea”, it should be “Human and Environmental Toxicology, University of Science and Technology, Daejeon, Republic of Korea”. Author Goo Yoon was erroneously assigned to affiliation 5. This has now been corrected by replacing affiliation 5 with affiliation 3 (“Department of Pharmacy, College of Pharmacy, Mokpo National University, Muan, Republic of Korea”) for author Goo Yoon. Subsequently, affiliation 6 has been renumbered as affiliation 5 for author Woo-Keun Kim.

The original article has been updated.

