# Supplementary material for: Licochalcone D reduces H2O2-induced SH-SY5Y cell neurotoxicity by regulating reactive oxygen species
Source: Front Pharmacol. 2025 Sep 18;16:1573882. doi: 10.3389/fphar.2025.1573882 (PMC12488560; doi:10.3389/fphar.2025.1573882)
Supplement: Supplementary file 1 [file DataSheet1.docx]

Supplementary Material


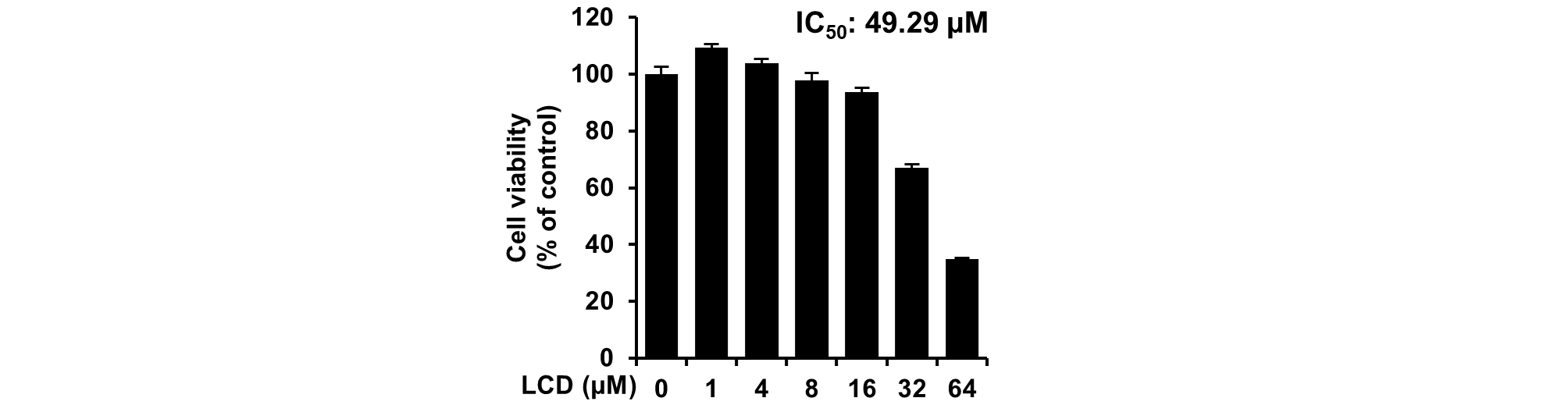


**Fig. S1. Effect of LCD on cell viability in differentiated SH-SY5Y cells.** SH-SY5Y cells were differentiated for five days and treated with LCD for 24 h. Cell viability was assessed via CCK-8 assay.


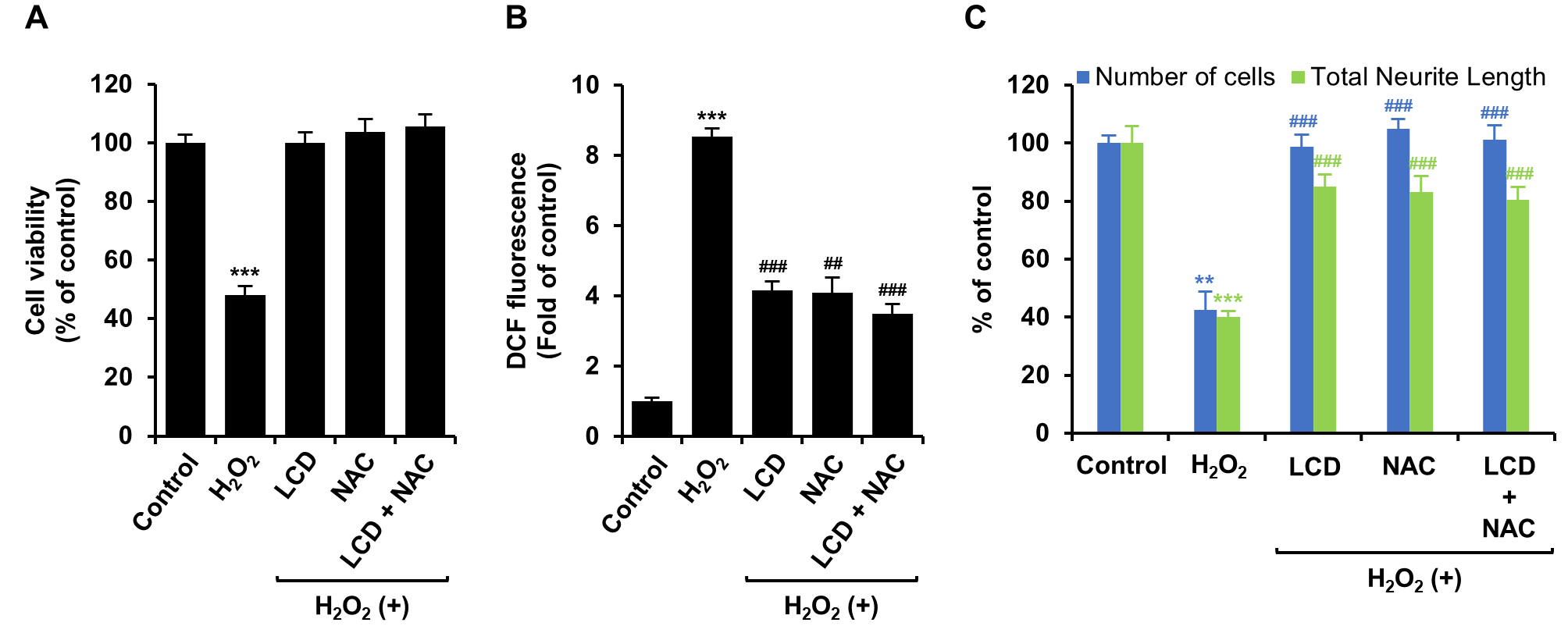


**Fig. S2. Effects of LCD and NAC co-treatment on H_2_O_2_-induced neurotoxicity in SH-SY5Y cells.** Differentiated SH-SY5Y cells were pre-treated with LCD (2 μM) and NAC (2 mM) for 3 h, followed by exposure to H_2_O_2_ (25 μM). (a) Cell viability was assessed by CCK-8 assay after 24 h of exposure. (b) Intracellular ROS levels were measured using DCFDA fluorescence after 3 h of pre-treatment and 1 h of H_2_O_2_ exposure. (c) For neurite outgrowth analysis, pre-treated cells were exposed to H_2_O_2_ for 24 h and were fixed and immunostained with β-III tubulin antibody, then with Alexa Flour 488-conjugated secondary antibody. Neurite outgrowth and nuclei were quantified using high-content screening analysis. The results are presented as mean ± SEM (*n* = 6). ***p* < 0.01, and ****p* < 0.001 vs the control group; ^##^*p* < 0.01, and ^###^*p* < 0.001 vs the H_2_O_2_-treated group.
